# Genetic association of the epidermal growth factor gene polymorphisms with peri-implantitis risk in Chinese population

**DOI:** 10.1080/21655979.2021.1983976

**Published:** 2021-10-20

**Authors:** Zhongfu Chang, Dandan Jiang, Shikun Zhang, Dongdong Pei, Zhirong Zhang, Lihua Zhang, Jianying Cai, Jun Cao

**Affiliations:** a Department of Stomatology, Shanghai Pudong New Area People’s Hospital, Shanghai China; b Department of Hemodialysis, Shanghai Pudong New Area People’s Hospital, Shanghai China

**Keywords:** Peri-implantitis, *egf*, polymorphism, genetic susceptibility

## Abstract

Peri-implant disease is an inflammatory disease and is related to genetic heterogeneity. Considering the genetic association of epidermal growth factor (*EGF*) gene polymorphisms with the susceptibility of periodontitis, its genetic association with peri-implantitis risk in a Chinese Han population was explored. Three hundred individuals who underwent dental implants were recruited, and divided into healthy implant group and peri-implantitis group. The genotype and allele distribution of *EGF* gene rs2237051 and rs4444903 polymorphisms were analyzed via direct sequencing and the frequencies were compared between the two groups using chi-square test. No significant difference was detected for the clinical information between healthy implant group and peri-implantitis group, including lifestyle habits platform type and position, peri-implant phenotype, brushing time, dental floss, and mouth washing frequencies. Individuals with peri-implantitis had poor periodontal status. The GG genotype and G allele of rs2237051 showed significant increasing trend in peri-implantitis group compared with the healthy implant group. Compared with the AA genotype carriers, rs2237051 GG genotype carriers showed lower risk to suffer from peri-implantitis (OR = 0.236, 95%CI = 0.089–0.624), and possessed low values of gingival index, plaque index and calculus index, peri-implant pocket depth (PPD) and clinical attachment level (CAL). But there was no significant difference for the rs4444903 genotype distributions between the case and control groups. In summary, EGF rs2237051 polymorphism showed close association with the genetic background of peri-implantitis. Rs2237051 GG genotype and G allele might be protective factors for the onset of peri-implantitis.

## Introduction

Peri-implant disease is an inflammatory disease occurring in the hard and soft tissues around the dental implant. It is one of the common complications after implant repair [1]. Peri-implantitis is the main cause of dental implant failure [2]. Periodontal tissue of the dental implant has a poor blood supply and weak resistance to inflammation. Therefore, once the occurrence of peri-implantitis, the inflammation progresses faster and more severely, which will eventually lead to the loss of the implant and dental implant failure. The biological process of peri-implantitis includes three stages: inflammatory response, connective tissue destruction and bone resorption [3]. In recent years, it has been observed that the occurrence of peri-implantitis is not uniformly distributed, showing the phenomenon of aggregation. It was considered to be related to genetic heterogeneity.

The pathogenesis and clinical manifestations of peri-implantitis are similar to those of periodontitis, and a history of periodontitis will increase the risk of peri-implantitis [4]. Studies have shown that gene polymorphisms of inflammatory cytokines affect the degree of inflammation in patients with periodontal bacterial infection, and also determine the susceptibility of individuals to periodontitis [5]. The occurrence of periodontitis is related to the genotype of patients. It has been reported that more than half of the onset of periodontitis patients can be attributed to genetic problems [6]. Consistently, multiple polymorphic sites in inflammatory cytokines-related genes, such as IL-1 and TNF-α, have been reported to play an important role in peri-implantitis [7,8].

Epidermal growth factor (EGF) is an original energy protein in human epidermal cells, which can promote cell proliferation and differentiation, enhance cell metabolism, and promote epidermal wound healing. Abnormal expression of *EGF* gene has been widely reported in patients suffering from periodontitis [9,10], which might be related to the increased activity of collagenase and gelatinase. *EGF* gene is located on chromosomal band 4q25-27 [11]. A number of single nucleotide polymorphisms (SNPs) have been identified in the gene, which may influence the function of the protein. Recently, genetic association of *EGF* gene polymorphisms with the susceptibility of periodontitis has been detected by Wang et al. [12] in a Chinese Han population. Rs2237051 and rs4444903 are two common SNPs of *EGF* gene, and show close association with genetic susceptibility of inflammatory diseases, including periodontitis [13,14]. However, the genetic association of *EGF* gene with Peri-implantitis has not been explored, which attracts our interest.

Therefore, in the present study, we explored the genetic association of *EGF* gene rs2237051 and rs4444903 polymorphisms with peri-implantitis risk in a Chinese Han population, in order to study the theoretical foundation for the mechanism of peri-implantitis. A total of 300 cases underwent dental implant were recruited, and divided into control and case group according to the peri-implantitis status. The rs2237051 and rs4444903 genotype and allele distributions in the case and control groups were compared, and their association with the periodontal status was also evaluated.

## Material and Methods

### Subjects

Three hundred cases who underwent dental implant were recruited in the case and control study, in which 150 patients suffered from peri-implantitis (identified as the case group) and the other 150 individuals were healthy implants (as the control group). All participants were aged from 24 to 60 years old. The inclusion criteria of the case group were as follows: 1) had no history of systemic disease or occlusal trauma; 2) presence of one or more implants with a minimum of 12 months loading period; 3) presence of bleeding on probing (with or without suppuration), with probing pocket depths more than 5 mm; 4) Radiographic sign of crestal bone loss in at least one area around an implant and the implant exposed at least two edges. The inclusion criteria of the control group were as follows: 1) presence of one or more implants with a minimum of 12 months loading period; 2) had no history of systemic disease or occlusal trauma; 3) had no radiographic signs of bone resorption; 4) had probing pocket depths less than 3 mm around any implant/teeth presented in their mouths.

This study design was designed under the approval of ethics committee of Shanghai Pudong New Area People’s Hospital. The sample collection of this study was based on ethics criteria of national human genome research. Written informed consent was obtained from each subject. All participants involved in this paper were Chinese Han population and not related by blood, which was determined by questionnaires given to participants.

### Clinical samples collection and DNA extraction

5 ml peripheral venous blood sample was collected from each subject, and anticoagulated by 0.5% EDTA (pH 8.0), separated into serum and hemocyte. The genomic DNA was extracted from the peripheral blood using TaKaRa Genome DNA Extraction Kit (Dalian Biological Engineering CO., LTD, China) and then stored at −20°C for standby application.

### Genotyping

The target fragments of *EGF* gene rs2237051 and rs4444903 polymorphisms were partially amplified using polymerase chain reaction (PCR). Primer Premier 5.0 was applied for the design of the primer sequences [15]. As shown in Table 1, all sequences were provided by Shanghai Sangon Biotech Co., Ltd. The PCR reactions were performed in a total volume of 25 μl, including 2 μl genomic DNA, 2 μl primer (1 μl each of upstream and downstream), 1.5 μl Mg^2+^, 2 μl dNTP, 0.3 μl Taq DNA polymerase, 2.5 μl 10× buffer and 14.7 μl ddH_2_O. The PCR procedures started with an initial degeneration 96°C for 5 min, followed by 32 cycles of 95°C for 30 s, annealing at 54°C for 30 s, 72°C for 30 s, and a final extension at 72°C for 10 min.

**Table 1.** Primer sequences of *EGF* gene rs2237051 and rs4444903 polymorphisms

Variations	Primer sequences
rs2237051	Forward 5ʹ-TTCAAGCCTTGTCCTTTCGT-3’
	Reverse 5ʹ-TTCAAAATCAGCAAAAGCATAAA-3ʹ
rs4444903	Forward 5ʹ-CATTTGCAAACAGAGGCTCA-3’
	Reverse 5ʹ-TGCTCTGGCTGACTTCACTG-3’ʹ

After purification, the amplified fragments were genotyped by automated DNA sequencing. The direct sequencing was done on an ABI 3730 DNA Analyzer (Applied Biosytems).

### Statistical analysis

The frequencies of both genotypes and alleles were counted, and the Hardy–Weinberg equilibrium (HWE) of each SNP was calculated to evaluate the representativeness of our study cohort. All data analysis was done in SPSS statistical software (IBM SPSS, V.23). Chi-square test and one-way ANOVA were used for the divergence analysis of categorical or continuous variables, respectively. *P* < 0.05 was considered to be statistically significant.

## Results

To explore the genetic association of *EGF* gene rs2237051 and rs4444903 polymorphisms with peri-implantitis risk, a total of 300 cases underwent dental implants were recruited. All cases were divided into control and case groups according to the peri-implantitis status. The rs2237051 and rs4444903 genotype and allele distributions in the case and control groups were compared, and their association with the periodontal status was also evaluated.

### Basic clinical information of the subjects

As shown in Table 2, there were 150 cases in each group. The control group consisted of 79 men and 71 women, with the mean age of 42.55 ± 6.93 years old. The case group included 83 men and 67 women, with the mean age of 43.37 ± 6.25 years old. The age and sex distribution showed no significant difference between the two groups (*P* > 0.05). The lifestyle habits, including smoking and drinking, were also recorded between the two groups and no significant differences were found (*P* > 0.05). It was observed that in patients with peri-implantitis, there are more cases that had the history of periodontitis, but the difference did not reach significant level in comparison with the healthy group (*P* > 0.05). Other basic information, including platform type and position, peri-implant phenotype, brushing time, dental floss, and mouth washing frequencies, were also compared, and no significant differences were found (all *P* > 0.05).

**Table 2.** Demographic and clinical information of the clinical subjects

Parameters	Healthy implants (n = 150)	Peri-implantitis (n = 150)	*p-*value
Age, (mean±SD)	42.55 ± 6.93	43.37 ± 6.25	0.298
Gender, n(%)			
Male	79(52.67)	83(55.33)	0.643
Female	71(47.33)	67(44.67)	
Alcohol consumption, n(%)			
Yes	60(40.00)	61(40.67)	0.906
No	90(60.00)	89(59.33)	
History of smoking, n(%)			
Yes	69(46.00)	68(45.33)	0.908
No	81(54.00)	82(54.67)	
History of periodontitis, n(%)			
Yes	68(45.33)	84(56.00)	0.065
No	82(54.67)	66(44.00)	
Platform type, n(%)			
External hex	70(46.67)	63(42.00)	0.353
Internal hex	22(14.67)	34(22.67)	
Morse cone	48(32.00)	45(30.00)	
Others	10(6.66)	8(5.33)	
Position, n(%)			
Anterior region	96(64.00)	81(54.00)	0.078
Posterior region	54(36.00)	69(46.00)	
Peri-implant phenotype, n(%)			
Thin	82(54.67)	70(46.67)	0.166
Thick	68(45.33)	80(53.33)	
Brushing daily, n(%)			
1–3 times	127(84.67)	129(86.00)	0.744
More than 3 times	23(15.33)	21(14.00)	
Dental floss daily, n(%)			
Yes	58(38.67)	62(41.33)	0.276
No	28(18.67)	18(12.00)	
Infrequent	64(42.67)	70(46.67)	
Mouth washing daily, n(%)			
Yes	44(29.33)	56(37.33)	0.339
No	34(22.67)	30(20.00)	
Infrequent	72(48.00)	64(42.67)	

Note: Continuous variable was expressed as mean ± standard deviation (SD) and compared via one-way ANOVA; Categorical variables were expressed as the number and percentage, total numbers in each category were compared using chi square test.

### Periodontal status of the study subjects

The periodontal status of the two groups was also recorded and analyzed. As shown in Table 3, individuals underwent peri-implantitis showed high levels of gingival index, plaque index, and calculus index compared with those healthy implants (*P* < 0.001). In addition, higher scores of peri-implant pocket depth (PPD) and clinical attachment level (CAL) were detected in peri-implantitis cases than those had the healthy implants (*P* < 0.001).

**Table 3.** Periodontal status of the studied population

Parameters	Healthy implants (n = 150)	Peri-implantitis (n = 150)	*p* value
Gingival index	0.53 ± 0.50	2.44 ± 0.52	< 0.001
Plaque index	0.77 ± 0.57	2.27 ± 0.61	< 0.001
Calculus index	0.14 ± 0.35	0.49 ± 0.59	< 0.001
PPD (mm)	1.87 ± 0.51	5.38 ± 0.77	< 0.001
CAL (mm)	1.39 ± 0.49	4.58 ± 0.74	< 0.001

Note: PPD, peri-implant pocket depth; CAL, clinical attachment level. Continuous variables were expressed as mean ± standard deviation (SD) and compared via one-way ANOVA.

### Correlation of EGF polymorphism with the susceptibility of peri-implantitis

As shown in Table 4, the genotype distributions of the two SNPs of *EGF* gene conformed to Hardy–Weinberg equilibrium (HWE) in the control group, indicating the study population has a similar genetic background to the Mendelian population. Frequencies of genotypes and alleles were counted and compared between the case and control groups. Higher frequency of rs2237051 GG genotype was detected in patients with peri-implantitis than those with healthy implants (*P* < 0.05), cases with GG genotype might have high predisposition of peri-implantitis (OR = 0.236, 95%CI = 0.089–0.624). Moreover, the G allele frequency increased significantly in peri-implants group (*P* < 0.05), and G allele demonstrated a high susceptibility to peri-implantitis (OR = 0.666, 95%CI = 0.474–0.938).

**Table 4.** Frequency distribution of *EGF* gene rs2237051 and rs4444903 genotype and allele in healthy implants and peri-implantitis groups

Genotype /Allele	Healthy implants n = 150 (%)	Peri-implantitis n = 150 (%)	χ^2^	*P* value		OR (95% CI)
rs2237051						
AA	57(38.0%)	69(46.0%)	-	-	1	
AG	72(48.0%)	75(50.0%)	0.381	0.537	0.861 (0.534 − 1.387)	
GG	21(14.0%)	6(4.0%)	9.421	0.002*	0.236 (0.089–0.624)	
A	186(62.0%)	213(71.0%)	-	-	1	
G	114(38.0%)	87(29.0%)	5.454	0.020*	0.666 (0.474–0.938)	
*P*^HWE^	0.819					
rs4444903						
GG	74(49.3%)	71(47.3%)	-	-	1	
AG	68(45.3%)	70(46.7%)	0.008	0.767	0.932 (0.585–1.486)	
AA	8(5.3%)	9(6.0%)	0.096	0.756	0.853 (0.312–2.333)	
G	216(72.0%)	212(70.7%)	-	-	1	
A	84(28.0%)	88(29.3%)	0.130	0.718	0.937 (0.658–1.335)	
*P*^HWE^	0.128					

Note: OR, odds ratio; CI, confidence interval; HWE, Hardy–Weinberg equilibrium. Categorical variables were expressed as the number and percentage, the difference between groups were compared using chi square test.

In terms of rs4444903, three genotypes (GG, AG, AA) were detected in the study population, but there was no significant difference for the genotype distributions between the case and control groups. Similar trend was also observed for the allele distributions (*P* > 0.05).

### The periodontal status of cases with different EGF gene polymorphisms

The periodontal status of cases with different genotypes of EGF gene polymorphisms was further analyzed. It was found that individuals carrying rs2237051 GG genotype possessed low values of gingival index, plaque index and calculus index, PPD and CAL, and the differences reached a significant level (*P* < 0.05, Table 5). The data illuminated that rs2237051 GG genotype was closely linked with the better periodontal status. Individuals carrying different rs4444903 genotypes showed no significant difference for the periodontal status (*P* > 0.05).

**Table 5.** The periodontal status of cases with different *CXCR2* gene polymorphisms

Parameters	rs2237051					rs4444903		
	AA	AG	GG	*p* value	GG	AG	AA	*p* value
Gingival index	1.65 ± 0.96	1.44 ± 1.15	0.96 ± 1.07	0.008	1.54 ± 0.97	1.43 ± 1.17	1.47 ± 1.28	0.726
Plaque index	1.75 ± 0.82	1.44 ± 0.99	0.89 ± 1.01	< 0.001	1.62 ± 0.83	1.41 ± 1.02	1.59 ± 1.28	0.158
Calculus index	0.40 ± 0.55	0.27 ± 0.49	0.18 ± 0.40	0.031	0.32 ± 0.53	0.30 ± 0.47	0.41 ± 0.71	0.667
PPD (mm)	3.89 ± 1.89	3.54 ± 1.91	2.81 ± 1.39	0.020	3.62 ± 1.97	3.63 ± 1.85	3.53 ± 1.37	0.975
CAL (mm)	3.40 ± 1.85	2.78 ± 1.60	2.11 ± 1.09	< 0.001	3.09 ± 1.93	2.93 ± 1.53	2.47 ± 1.07	0.338

Note: PPD, peri-implant pocket depth; CAL clinical attachment level. Continuous variable was expressed as mean ± standard deviation (SD) and compared via one-way ANOVA.

## Discussion

With the development of social economic level and the improvement of dental implant technology, dental implant has become the first choice for people to repair partial or total dental loss. Peri-implantitis is the leading cause of implant loosening and failure, with an incidence of 28–56% [16]. There are many predisposing factors of peri-implantitis, including age, sex, poor oral hygiene, smoking habits, a history of periodontitis, and alcohol intake. In the current study, the predisposing factors of the two study groups were compared, and no significant difference was detected. It is believed that the case and control groups are comparable. After excluding the influence of these factors, it was clinically found that most of the peri-implantitis occurred in specific populations [17]. Therefore, host factors may influence the occurrence of peri-implantitis. Recent studies have shown that genetic factors are also very important for the occurrence of peri-implantitis [18,19]. Genetic predisposition is responsible for the increased risk of peri-implantitis in implant patients [20]. In recent years, a large number of genes have been identified to be linked with the susceptibility of peri-implantitis, such as *IL-1, RANKL* gene [21,22].

In the present study, two common SNPs of the *EGF* gene were selected, and their genetic association with the onset of peri-implantitis was explored. A close association was detected between *EGF* gene rs2237051 with peri-implantitis occurrence. EGF is a cellular factor that performs a key role in many physiological and pathological processes [23]. Several SNPs have been identified in the *EGF* gene, which affects the expression level of EGF protein, and then participates in the regulation of body life activities. In a case-control study, Wang et al. investigate the connotation between *EGF* polymorphisms and generalized aggressive periodontitis (GAgP) [13]. Elevated serum levels of EGF are detected in GAgP patients, and *EGF* rs2237051 SNP is associated with the risk of GAgP. The rs2237051 AA genotype carriers perform elevated risk for GAgP, and the serum EGF levels are detected to be increased for patients carrying AA genotype. The pathogenesis and clinical manifestations of peri-implantitis are similar to those of periodontitis, and a history of periodontitis may increase the risk of peri-implantitis. In consideration of the close relationship between rs2237051 and periodontitis, whether rs2237051 polymorphism is correlated to the genetic background of peri-implantitis was explored.

A total of 300 cases who underwent dental implants were recruited in the case and control study, in which 150 patients suffered from peri-implantitis and the other 150 individuals were healthy implants. The frequencies of rs2237051 genotypes and alleles in the case and control groups were counted and compared, and the distribution of rs2237051 genotypes showed a remarkable difference between peri-implantitis and healthy implants. It was detected that the rs2237051 GG genotype carriers were 76% less likely to suffer from peri-implantitis than those carrying the AA genotype. Previously, in cases with GAgP, rs2237051 AA genotype is suggested to elevate the level of serum EGF [13]. EGF plays a crucial role in inflammation and tissue repair, serving as an important regulator in the process of periodontitis and bone destruction [24,25]. It is also involved in the regulation of tissue immune response, inhibits collagen synthesis by fibroblasts and affects osteoclast bone resorption, which plays an important role in periodontitis [24,26,27]. High levels of EGF can be detected in patients with oral diseases and are believed to be closely related to the occurrence and development of periodontitis [28]. Consideringly, it was deduced that rs2237051 AA genotype may promote the expression level of EGF in cases that underwent dental implant, further contribute to inflammation and the risk of peri-implantitis. Furthermore, the periodontal status of cases with different genotypes of EGF gene polymorphisms was further analyzed. It was found that individuals carrying rs2237051 AA genotype possessed high values of gingival index, plaque index and calculus index, PPD and CAL. The data illuminated that rs2237051 AA genotype was closely linked with the poor periodontal status. In addition, the G allele carriers of rs2237051 had a decreased risk of peri-implantitis. Recently, the positive association between rs2237051 polymorphism and periodontitis has been replicated in several other populations in China [12,29], which supported our present findings.

Rs4444903 is identified to be a functional variation carrying an A-to-G mutation (−61A > G) at the 5ʹ- untranslated region (UTR) of the *EGF* gene, leading to the upregulation of the gene levels [30]. It has been identified to influence the susceptibility of several cancers, such as hepatocellular carcinoma, colorectal cancer [31,32]. However, no correlation was found between rs4444903 genotype and the occurrence of peri-implantitis. This may be related to the small number of samples included in this study, further studies with a larger population should be performed to verify the present findings. In addition, this study is only a basic analysis of the clinical findings, and further research is needed to determine the possible mechanisms.

The current study has limited evidence in terms of the association between *EGF* polymorphisms and peri-implantitis susceptibility, due to their small number of patients and lack of cross-ethnic comparisons. Follow-up studies with more sample sizes and research indicators are needed.

### Conclusion

In conclusion, this study preliminary proposed the relationship between *EGF* gene polymorphism and susceptibility to peri-implantitis, *EGF* rs2237051 mutation is associated with the occurrence of peri-implantitis. The findings will be helpful for the elucidation of the genetic background of peri-implantitis. It is known that the susceptibility to peri-implantitis is not limited to a single factor, but appears to be associated with multiple factors. Therefore, actively controlling the susceptibility factors in patients can reduce the incidence of peri-implantitis and improve the success rate of implantation therapy.

## Article highlight

Rs2237051 GG genotype and G allele protect against the onset of peri-implantitis.

Rs2237051 GG genotype was closely linked with the better periodontal status.

*EGF* rs2237051 polymorphism was related to the genetic background of peri-implantitis.
